# A Study of Natural Radioactivity Levels and Radon/Thoron Release Potential of Bedrock and Soil in Southeastern Ireland

**DOI:** 10.3390/ijerph18052709

**Published:** 2021-03-08

**Authors:** Mirsina Mousavi Aghdam, Quentin Crowley, Carlos Rocha, Valentina Dentoni, Stefania Da Pelo, Stephanie Long, Maxime Savatier

**Affiliations:** 1 Department of Civil and Environmental Engineering and Architecture, University of Cagliari, 09123 Cagliari, Italy; vdentoni@unica.it; 2 Department of Geology, School of Natural Sciences, Trinity College, D02PN40 Dublin, Ireland; crowleyq@tcd.ie; 3 Biogeochemistry Research Group, School of Natural Sciences, Trinity College, D02PN40 Dublin, Ireland; rochac@tcd.ie (C.R.); savatiem@tcd.ie (M.S.); 4 Department of Chemical and Geological Sciences, University of Cagliari, 09042 Cagliari, Italy; sdapelo@unica.it; 5 Environmental Protection Agency of Ireland, D14YR62 Dublin, Ireland; S.Long@epa.ie

**Keywords:** radon and thoron exhalation rates, airborne radiometric, radiation risk, geological combination and soil type

## Abstract

Radon (^222^Rn) and thoron (^220^Rn) account for almost two-thirds of the annual average radiation dose received by the Irish population. A detailed study of natural radioactivity levels and radon and thoron exhalation rates was carried out in a legislatively designated “high radon” area, as based on existing indoor radon measurements. Indoor radon concentrations, airborne radiometric data and stream sediment geochemistry were collated, and a set of soil samples were taken from the study area. The exhalation rates of radon (E^222^_Rn_) and thoron (E^220^_Rn_) for collected samples were determined in the laboratory. The resultant data were classified based on geological and soil type parameters. Geological boundaries were found to be robust classifiers for radon exhalation rates and radon-related variables, whilst soil type classification better differentiates thoron exhalation rates and correlated variables. Linear models were developed to predict the radon and thoron exhalation rates of the study area. Distribution maps of radon and thoron exhalation rates (range: E^222^_Rn_ [0.15–1.84] and E^220^_Rn_ [475–3029] Bq m^−2^ h^−1^) and annual effective dose (with a mean value of 0.84 mSv y^−1^) are presented. For some parts of the study area, the calculated annual effective dose exceeds the recommended level of 1 mSv y^−1^, illustrating a significant radiation risk. Airborne radiometric data were found to be a powerful and fast tool for the prediction of geogenic radon and thoron risk. This robust method can be used for other areas where airborne radiometric data are available.

## 1. Introduction

Exposure to natural background radiation (especially radon) is an important environmental issue. As a consequence (and in accordance with EU regulation), most of the developed member states have active radon monitoring programs as part of their National Action Plans. Indoor radon and gamma radiation from primordial radionuclides (i.e., decay products of ^238^U and ^232^Th and ^40^K) are responsible for most of the average annual dose absorbed by humans [1,2]. Radon, a natural radioactive gas, is produced via the decay chain of ^238^U, ^232^Th and ^235^U. The most abundant radioisotopes of radon are ^222^Rn (from the decay chain of ^238^U-hereafter called radon) and ^220^Rn (from the decay chain of ^232^Th-hereafter called thoron) [3]. Inhalation of radon and subsequent emission of radioactive progeny (i.e., ^218^Po, ^214^Po, ^214^Pb, ^214^Bi) can cause the irradiation of lung and bronchial tissues, which is associated with a higher risk of developing lung cancer [2]. In other words, both instantaneous and long-term exposure to high radon concentrations play a crucial role in the risk of developing lung cancer.

Following several observations in homes affected by high indoor radon concentration, gas flux from bedrock and soil hosting radium-bearing minerals were considered the major source of radon, while building materials or well waters were noted as possible additional secondary issues [4]. Radon in soil and rock may migrate through soil pores, faults and fractures in bedrock by gas-phase diffusion and advection into the shallow region between the soil and the building foundation or basement [3]. The presence of carrier gases, such as CO_2_, in the soil profile may facilitate the migration of radon to ground level. Joints, gaps or fractures in the building structures can then provide radon gas entry pathways to indoor spaces [5]. Indoor radon levels in neighboring dwellings can vary significantly due to the complicated influence of building characteristics, meteorological parameters and living habits [6]. Nevertheless, geological characteristics remain the primary controlling factors of the risk assessment protocol [7]. By mapping the Geogenic Radon Potential (GRP) it is possible to identify radon-prone areas (RPA) [8], i.e., areas where indoor radon concentration is likely to be higher than the national average [9]. A GRP map is typically prepared exclusively based on geological information (e.g., radiometric and geochemical data, lithological types, U and Ra content, soil-gas radon, and soil permeability) [3,10,11,12]. Among the data mentioned, airborne gamma-ray spectrometer surveys can be efficiently utilized to provide high-resolution geogenic radon and thoron maps expressed in terms of equivalent concentrations of their parent nuclides; uranium-238 (eU, estimated from ^214^Bi) and thorium-232 (eTh, estimated from ^208^Tl), respectively [13].

The goal of this research is to identify radon and thoron (i.e., ^222^Rn and ^220^Rn) prone areas (RPA) within southeastern Ireland and develop a methodology that can be applied to map RPAs in other areas where similar data sets are available. To achieve this, a set of data, including indoor radon concentrations, airborne radiometric data, geochemistry of stream sediments and measurements of ambient dose rate, together with laboratory testing of ^222^Rn and ^220^Rn exhalation rates, were employed to classify the radon and thoron release potential. First, the above-mentioned quantities were classified based on (a) geological boundaries and (b) soil type variations. Secondly, the spatial correlation between radon-related variables was studied using a Pearson’s matrix for both classification systems, in order to understand which criterion (geological boundaries or soil types) is the better classifier of radon and thoron release/exhalation potential. At the final step of this research, the feasibility of reproducing radon/thoron exhalation rate maps using radiometric data (i.e., equivalent ^238^U and ^232^Th concentrations) was evaluated by the progression of the correlation between measured ^222^Rn and ^220^Rn exhalation rates and the available radiometric data for the location of sampling points. These maps can potentially be reproduced for other parts of Ireland or other countries where airborne radiometric, geological and soil data are available.

## 2. Materials and Methods

### 2.1. Geological Setting and Soils of the Study Area

**Figure 1:**
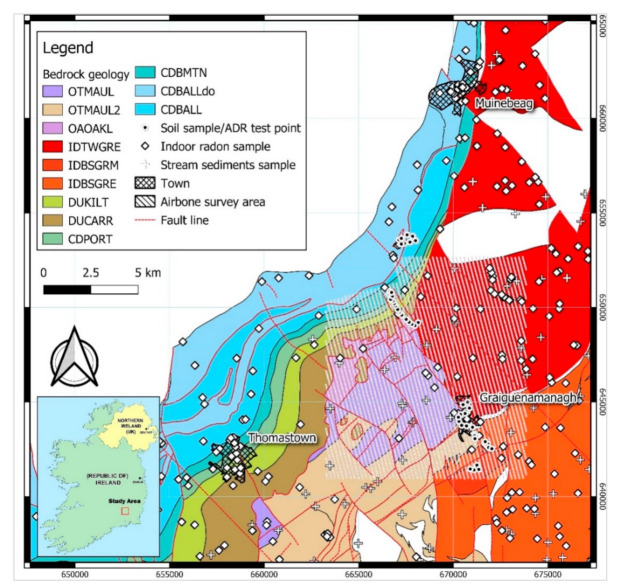
Bedrock geology (at 1:100,000 scale-Irish Transverse Mercator (ITM)) of the study area with location of the sampling and airborne survey sites; OTMAUL (dark blue-grey slate, phyllite and and schist), OTMAUL2 (Dark grey semi-pelitic, psammitic schist), OAOAKL (Green, red-purple, buff slate, siltstone), IDTWGRE (Pale, fine to coarse-grained granite- Tullow type Equigranular Granite), IDBSGRM (Granite with microcline phenocryst), IDBSGRE (Pale, fine to coarse-grained granite-Blackstairs type, Equigranular Granite) DUKILT (Yellow and and red sandstone and and green mudstones), DUCARR (Red, brown conglomerate and and sandstone), CDPORT (Sandstone, shale and and thin limestone), CDBMTN (Limestone and dark-grey calcareous shale), CDBALLdo (Dolomitized dark-grey muddy limestone), CDBALL (Dark muddy limestone, shale). Base map obtained from https://www.gsi.ie/en-ie/data-and-maps/Pages/Bedrock.aspx (accessed on 26 November 2019).

The study area is located about 130 km SW of Dublin, in southeastern Ireland, in County Kilkenny on the border with County Carlow. It includes the towns of Graignamanagh, Thomastown and Muine Bheag. The geology of the study area is principally composed of Lower Palaeozoic, Devonian and Carboniferous lithologies, with extensive and spatially variable overlying subsoil deposits. According to Geological Survey Ireland (GSI) resources (https://www.gsi.ie/en-ie/data-and-maps/Pages/Bedrock.aspx) (accessed on 26 November 2019) and the 1:100,000 bedrock geology map of Ireland (Figure 1), the study area comprises 12 geological formations. Lower Palaeozoic strata outcrop at the eastern and southeastern part of the area, in which the main deposits comprise metasedimentary (phyllites and schists of the Maulin Formation). These are intruded by plutonic igneous rocks (Caledonian Leinster Granite). Devonian sandstones and conglomerates overly an offshoot of the Leinster Granite. Lower Carboniferous sedimentary rocks comprise a series of sandstones, siltstones, and mudstones in the southwestern part of the study area. The Sub-Waulsortian Limestones, namely the Ballymartin and Ballysteen Formations, mainly comprise bioclastic limestones [14], and conformably overlie Lower Carboniferous Sandstones and Shales. The Oakland Formation (OAOAKL) contains the Red-purple/green laminated siltstones and shales, green greywackes, laminated dark grey, green and buff shales and fine siltstones. However, he distinctively laminated and variegated siltstones and shales are the dominant lithology and occur in horizons 10–12 m thick [15]. 

**Figure 2:**
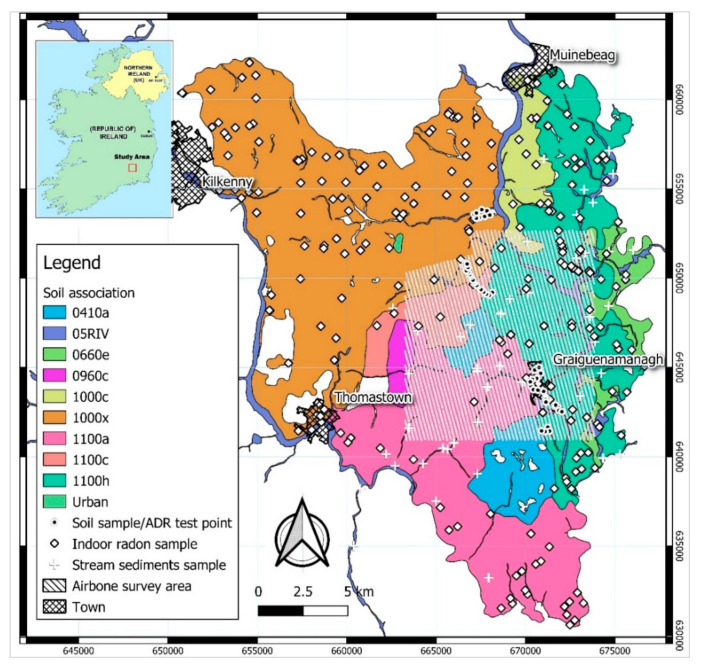
Soils map (at 1:250,000 scale Irish Transverse Mercator (ITM)-) of the study area with location of the sampling and airborne survey sites; 0410a (Peat over lithoskeletal acid igneous rock), 05RIV (River alluvium), 0660e (Coarse loamy drift with igneous and metamorphic stones), 0960c (Fine loamy over mudstone, shale or slate bedrock), 1000c (Fine loamy drift with limestones), 1000x (Fine loamy drift with limestones and substrate of siliceous stones), 1100a (Fine loamy drift with siliceous stones), 1100c (Coarse loamy drift with siliceous stones), 1100h (Coarse loamy drift with igneous and metamorphic stones). Base map obtained from http://gis.teagasc.ie/soils/map.php (accessed on 12 December 2019).

Soils in any area are the result of the interaction of various factors, such as parent rock or sediment material, climate, vegetation, and human activities. The soil classification map (Figure 2) (http://gis.teagasc.ie/soils/map.php) (accessed on 12 December 2019) identifies ten soil associations within the study area. The parent material of soils in the study area consists of surficial glacial drift with a considerable variation in geological composition, physical constitution and thickness. The dominant soil type is Grey Brown Podzolic which is deep and has a medium-heavy texture. They are well-drained soils derived from calcareous drift composed mainly of limestone with some shales and sandstone. Lighter, shallower soils are found on the river banks located in the area (e.g., Barrow river), derived from alluvial deposits, i.e., coarse-textured gravels and sands (05RIV, Figure 2) [16].

Previous studies have shown that some soils and rocks (e.g., granites, shales, psammites, semipelites, and meta-limestones) may be associated with higher levels of radon [17,18]. As it was discussed in previous paragraphs, outcrops of some of these geologies can be found in the study area. Moreover, traces of autunite (Ca(UO_2_)2(PO_4_)2·10–12H_2_O), a uranium-bearing mineral have been observed during geological explorations in Ordovician sediments within the area. According to such observations, it was deemed necessary to evaluate natural radioactivity levels and radon release potentials of the area under examination.

### 2.2. Collection and Management of Available Data

The rock/soil material is spatially heterogeneous even at the relatively small scale of tens of meters, therefore a statistical approach is necessary to characterize particular rock/soil types [19]. In this study, different rock and soil types were characterized in terms of potential for radon exhalation following two steps. In the first step, the vectorized version of bedrock geology map and soil associations were utilized in open-source QGIS Desktop 3.16.2 software-www.qgis.org (The Open Source Geospatial Foundation. Chicago, IL, USA) (accessed on 21 February 2020) and the mean value of radon-related quantities like indoor radon concentration, airborne radiometric survey data (i.e., equivalent uranium, equivalent thorium and potassium), soil geochemistry, ambient dose rates and radon exhalation rate were calculated for different bedrock geologies and soil types. In the second step, the correlation coefficients between the calculated mean values were evaluated using Pearson’s correlation matrix.

#### 2.2.1. Indoor Radon Data

In the process of mapping indoor radon risk, an important step is to define geological units that are associated with indoor radon concentration in a significant way [20]. As a part of the Irish national survey of indoor radon, the Environmental Protection Agency (EPA) of Ireland has collected indoor radon data measured over a minimum period of three months using passive alpha track detectors (CR-39) [21,22]. A total number of 2886 geo-referenced indoor radon data points were available for the study area under consideration, where high levels of indoor radon (i.e., higher than the Irish national reference level, 200 Bq m^−3^) were observed [23].

#### 2.2.2. Airborne Radiometric Survey

The Tellus programme managed by GSI involves (a) an airborne geophysical survey and (b) a ground-based geochemical survey of soil, stream water and stream sediment. The airborne radiometric survey is carried out by using a low-flying aircraft (flying at 60 m in rural areas and 240 m in urban areas). The data are recorded by a 256-channel gamma spectrometer (Exploranium GR820) covering 0.3–3 MeV [24]. Data are integrated over flying distances of about 50 m. Potassium (^40^K), equivalent uranium (eU, estimated from ^214^Bi) and equivalent thorium (eTh, estimated from ^208^Tl) were measured as a result of the surveys and finally, the recorded values were corrected for cosmic radiation, height attenuation and removing aircraft (i.e., the aircraft electrical devices can cause periodic or constant noise, the aircraft effect is removed in real-time during the survey, using the compensation data) [25]. Further information regarding the airborne radiometric data correction procedures has been published elsewhere [24,26,27].

It is noteworthy that airborne radiometric data from the Tellus programme is available for most of Ireland, and the entire island of Ireland is expected to be covered by 2023. Results from merged surveys are available on the Tellus website (www.tellus.ie) (accessed on 13 January 2020).

Some countries have employed radiometric data to produce geogenic radon potential maps [18,28]. In this study, a statistical approach was employed for studying the radon release behavior of different rocks and soils. The mean values of radioelement concentrations and air absorbed dose rates (ADR_air_) for different geological combinations and soil types were calculated accordingly. The classifications for the study area were derived from approximately 9400 airborne survey data points. 

#### 2.2.3. Geochemistry of Stream Sediment Samples

Both stream sediment and topsoil geochemistry can be useful for radon and even thoron potential mapping. Due to the short radioactive half-life of ^220^Rn (55.6 s), the diffusion length of ^220^Rn is only a few centimeters. Therefore, topsoil properties (e.g., soil water saturation, soil temperature and soil texture) can have a greater effect on the surface exhalation rates of thoron. [29,30] For mapping purposes, topsoil geochemical data is preferable as this type of data can better represent radon characteristics of the overlaying environment [30]. Stream sediment data, grouped by bedrock superficial geology classifications, was investigated concerning radon potential in Scotland [7] and it was found that indoor radon correlates most strongly with U, followed by Rb, K, Y, La, and Zr in stream sediments.

According to the Tellus programme, the GSI has collected soil and stream samples at a density of approximately one sample every 4 km^2^. Through multi-element laboratory analyses using X-ray fluorescence spectroscopy (XRF), geochemical data are provided for 55 elements [31]. At the time of writing, there are no topsoil geochemistry data available for the study area. However, approximately 500 data points on stream sediment geochemistry are available for the different bedrock geologies of the study area. The average K_2_O, U, Th, Zr, Y, CaO, SiO_2_, Al_2_O_3_, MgO, and Fe_2_O_3_ were calculated for each geological formation and soil category.

### 2.3. Measurements and Instruments

#### Ambient Dose Rate (ADR) Mapping and 222Rn/220Rn Exhalation Rates

In situ soil gas radon measurements performed in various lithological types can give the first radon characterization of an area; this approach is considered to be the most appropriate to estimate the geogenic radon potential [32,33]. However, laboratory testing of exhalation rate is sometimes preferred since in situ determinations of radon fluxes from the ground are actually time-consuming and affected by large uncertainties, due to changing physical conditions such as soil moisture, temperature and pressure gradients [34].

**Figure 3:**
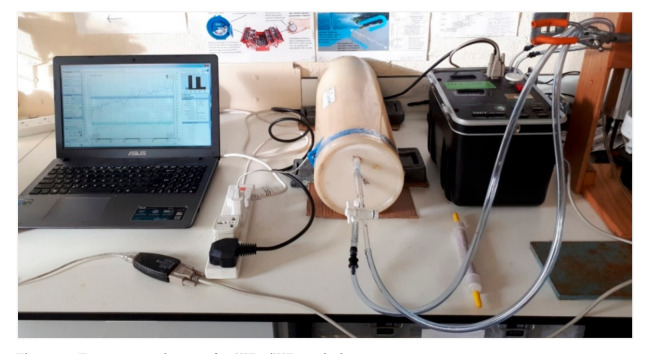
Experimental setup for ^222^Rn/^220^Rn exhalation rate measurements.

**Figure 4:**
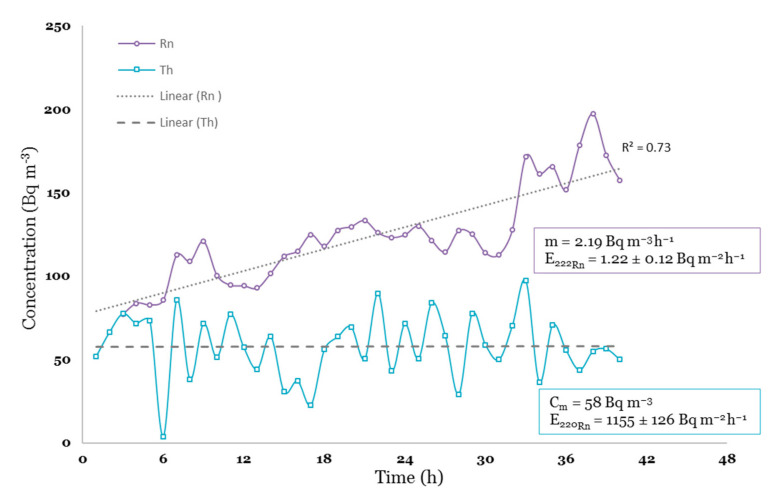
^222^Rn/^220^Rn concentrations as a function of time inside the accumulation chamber and calculated radon and thoron exhalation rates from the surface of a soil sample.

Integration of laboratory radon and thoron exhalation data with ground gamma radiation mapping has been successfully utilized to study the source of natural radiation and characterize the radon release potential of a volcanic area near Rome [34]. A similar approach has been adopted in this study to estimate the radon release potential of different rock and soil types, whereby 45 soil samples were taken at a depth of 50 cm below the surface using an auger sampler. The ambient gamma dose rate (μSv h^−1^) at 1m above the ground level was also measured at the same sampling point by using a handheld radio dosimeter (i.e., Radex RD1008, calibrated by the manufacturer). The recorded values were georeferenced and introduced to open source QGIS Desktop 3.16.2 software-www.qgis.org (The Open Source Geospatial Foundation. Chicago, IL, USA) (accessed on 21 February 2020). The soil samples were then oven-dried and sieved to 2 mm. About 500 g of each sample was introduced to a 7-L sealed accumulation chamber connected to a RAD7 radon monitor which was pre-calibrated in a radon standard chamber via an annual inspection and calibration (Billerica, MA 01821-2812, USA) (Figure 3). During the sample measurements inside a sealed chamber, the top caps of sample containers were left open to enable the exhalation of radon from one surface of the sample. A small fan was mounted inside the chamber to increase the circulation rate and ensure the best possible homogeneity of the enclosed air. ^222^Rn and ^220^Rn activities within the closed air loop were measured at 1-h cycles for about 36 h for each soil sample. Measurement time depended on the alignment of the recorded ^222^Rn data points and the time required to reach the equilibrium ^220^Rn concentration (see Figure 4). 

Considering Equations (1) and (2) [34] the exhalation rates of ^222^Rn (E^222^_Rn_, Bq m^−2^ h^−1^) and ^220^Rn (E^220^_Rn_, Bq m^−2^ h^−1^) were calculated by extrapolating the slope of the growth curve (m) (Bq m^−3^ h^−1^) and the equilibrium ^220^Rn concentration (C_m_) (Bq m^−3^), respectively. Figure 4 shows an example of ^222^Rn/^220^Rn concentrations as a function of time and the derived exhalation rates.

(1) $$E_{222\mathrm{Rn}}=\frac{(m+\lambda_{222}\times C_{0})\times V}{S}$$

(2) $$E_{220\mathrm{Rn}}=\frac{\lambda_{220}\times V_{0}}{S}\frac{C_{m}}{e^{-\lambda_{220}\times (V_{1}/Q)}}$$

where *λ*_222_ and *λ*_220_ are ^222^Rn and ^220^Rn decay constants (h^−1^), *C*_0_ is the initial radon concentration (Bq m^−3^), *V* is the free total volume of the analytical system (m^3^), *S* is the surface of the sample, *V*_0_ and *V*_1_ (m^3^) are the free volume of the accumulation chamber and the volume between the outflow of the accumulation chamber and the inflow of the radon monitor, respectively. *Q* (39 L h^−1^) is the flow rate in the system. 

Furthermore, to estimate the radiation risk to the public, the annual effective dose rate (AEDR) in mSv y^−1^ was calculated from the air absorbed dose rate (ADR_air_, nGy h^−1^) using Equation (3) as follows [35]:

(3) $$AEDR={\mathrm{ADR}}_{\mathrm{air}}\times 8760\times 1.0\times 0.7\times 10^{-6}$$

where the factor of 8760 represents the number of hours (h) in a year of 365 days and occupancy of unity has been applied. The conversion means that the airborne dose rate values of 10, 100 and 1000 (nGy.h^−1^) produce equivalent AEDR estimates of 0.061, 0.61 and 6.1 mSv, respectively, for a unitary indoor-outdoor exposure. The worldwide average AEDR is 0.48 mSv (78 nGy.h^−1^) with the results for individual countries being generally in the range 0.3–0.6 mSv [35]. 

## 3. Results

### 3.1. Grouping Based on Geological Bedrock Borders

As shown in Table 1, the highest values of geometric mean indoor radon (366 Bq m^−3^) and mean values of air potassium (5.15%), equivalent uranium and thorium (6.17 and 18.47 mg kg^−1^, respectively), air absorbed gamma dose rates (ADR_air_) (178.43 nGy h^−1^) and radon exhalation rates (1.52 Bq m^−2^ h^−1^) were found in areas underlain by granites with microcline phenocrysts (geocode: IDBSGRM). Granites are typically known as lithologies associated with higher radionuclide concentration [36]; however, there are significant variations of natural radioactivity levels in different granite types. The variations are mainly due to the different levels of accessory minerals such as orthite or allanite, monazite, zircon, apatite, and titanite concentrated in granitic rocks [37]. Other affecting parameters include hydrogeological processes causing ^226^Ra solution and precipitation (the most important parameters), geological phenomena such as faults, hydrothermal alterations and weathering conditions [38]. In this research two other types of granite (i.e., pale and fine to coarse-grained equigranular granites of Tullow and Blackstairs type (geocode: IDBSGRE and IDTWGRE, respectively) were also studied. Although these two units have lower levels of radon-related values (i.e., indoor radon, eU and U) compared to granites with microcline phenocryst, on average soils spatially associated with them exhibited enhanced levels of radon exhalation rates when compared with most other (non-granitoid) lithological types. Moreover, the green red-purple buff slates and siltstones of the Oakland’s formation show the second highest values among most of the radon-related values. This can be explained by the fact that some types of sedimentary rocks (e.g., phosphates, reworked igneous or magmatic clastic rocks [17]) may contain organic matter and silt-clay size mineral grains that cations of potassium, uranium and thorium can be placed into them [39]. The mean values of airborne eTh, eU and K concentrations are higher than the world average values of 45 Bq kg^−1^ (11.08 mg kg^−1^) for ^232^Th, 33 Bq kg^−1^ (2.67 mg kg^−1^) for ^238^U, and 412 Bq kg^−1^ (1.32 %) for ^40^K [35,40]. The mean value of calculated air absorbed dose rates is about 2 to 3 times the reported population-weighted mean value of 58 nGy h^−1^ for the typical area [35]. Table 2 shows the mean values of stream sediment geochemical data grouped by bedrock superficial geology, which were investigated for any correlations with radon-related variables. Among the elements analyzed, U and Th are considered as the most important ones since they are mostly used in studies related to the assessment of radon potential [7,34]. 

As anticipated, the highest U and Th activities correspond to the pale and fine to coarse-grained equigranular granites of Tullow and Blackstairs type. This correlation again strongly indicates the influence of a granitic protolith on radionuclide concentrations. At the time of writing, no stream sediment data were available for microcline phenocryst-bearing granites from the study area. A simple comparison between U and Th from stream sediments with eU and eTh from the radiometric survey for larger spatial areas shows that for most of the lithological types the activity of radioelements determined by XRF is lower than values determined by air-borne radiometric gamma. This can be due to the mobilization of radionuclides through aqueous solutions. The difference between datasets is more obvious for U than for Th. U forms soluble salts, which can be transported in river water. Conversely, Th will not easily dissolve and be transported by water during the weathering process [41]. On average K_2_O values are less than K_air_ activity concentration, which can be due to the adsorption of ^40^K in water [18]. Table 3 expresses the Pearson’s correlation coefficients (PCC) of indoor radon, airborne radiometric, geochemical data, ambient gamma dose rate and radon exhalation rates for geological combinations. It indicates that indoor radon is most strongly related to eU (from radiometric surveys) (PCC = 0.77) and U (from geochemical analysis) (PCC = 0.84). There is also a good correlation between radon exhalation rates and indoor radon (PCC = 0.62) and radon exhalation rates with eU (PCC = 0.69). These results are in agreement with previous studies [42,43]. 

There is a close correlation between the broad distributions on the radiometric and the ambient dose rates. This correlation is particularly strong between K_air_ and the ambient dose rate. The average ambient dose rates recorded by handheld radiometer are also correlated with air absorbed dose rates. This means that handheld radiometers can give a fast evaluation of gamma dose rate levels in an area. However, the values obtained by handheld radiometer are higher than radiometric air absorbed dose rates. This can be justified by the fact that no corrections were applied to the recorded gamma values by handheld radiometer and therefore, atmospheric radiation and cosmic radiation may probably have influenced ADR values. In the case of stream sediment geochemical data, positive correlations were found between U and Th and also between K_2_O, Al_2_O_3_, and Y. The good negative correlations are obtained for U and Th with MgO and also K_2_O with CaO. It worth noting that using the categorization of data based on geological combinations, there is almost no correlation between thoron exhalation rates, Th and eTh. This may be because of the short diffusion length of ^220^Rn and the depth to bedrock so that it would be unlikely for thoron to escape from the bedrock to the upper soil and into the atmosphere 

### 3.2. Grouping Based on Soil Type Variations

Table 4 reports the summary of indoor radon, airborne radiometric and radon/thoron exhalation rates statistics for different soil types. Among the soils tested, the peat over lithoskeletal acid igneous rocks (0410a) and coarse loamy drift with siliceous stones (1100c) showed the highest equivalent thorium concentration (16.22 mg kg^−1^) and thoron exhalation rate (2138 Bq m^−2^ h^−1^), respectively. The summary of soil geochemistry for grouped soil associations is expressed in Table 5. Table 6 shows Pearson’s correlation matrix of indoor radon, airborne radiometric, geochemical data, gamma dose and radon exhalation rates categorized based on soil variations. As can be understood from Table 4 and according to the weak correlation coefficients reported in Table 6, it was difficult to distinguish between radon-related characteristics (i.e., GM_Rn_, eU, E_222Rn_, and U) of different soil types. Comparing the correlation coefficients represented in Table 3 and Table 6, it is understood that although there are good correlations between radon exhalation rate and eU and between eU and U in the soil classification system, bedrock geology is a better classifier for ^222^Rn-related parameters when compared to the soil variations. On the other hand, based on the soil classification system, significant positive correlations were found between thoron exhalation rates (E_220Rn_), eTh (PCC = 0.62) and also Th with eTh (PCC = 0.83). Thoron exhalation rate depends on the thorium content of topsoil. Correlation between (E_220Rn_) and eTh, expresses that the eTh could be an indicator of soil thorium content. However, as mentioned earlier in this section, the soil that contains the highest equivalent thorium contents (0410a) is not essentially the one with the thoron highest exaltation rate. This may be because thoron exhalation rate can be also influenced by other parameters like topsoil physical properties.

Higher correlation coefficients for radon-related variables (i.e., indoor radon, eU, U and radon exhalation rate) in geological grouping systems and the good correlation of thoron-related values (i.e., eTh, Th and thoron exhalation rate) in the soil classification system can be explained by differences in ^222^Rn and ^220^Rn’s half-lives. Radon has a longer half-life and therefore can travel longer distances and be originated from deeper sources, in this case, lithology is the better predictor of the range of radon activities. However, as thoron decays quickly, the measured thoron in the ground level should be originating from shallow soil therefore and in this case, soil type variation better classifies thoron behaviors. Similar to the classification system based on geological combinations, the soil type grouping method identified positive correlation coefficients between U and Th and also K_2_O with Al_2_O_3_ and Y.

### 3.3. Estimation of Annual Effective Dose Rate and Radon Release Potential Based on Airborne Radiometrics

**Figure 5:**
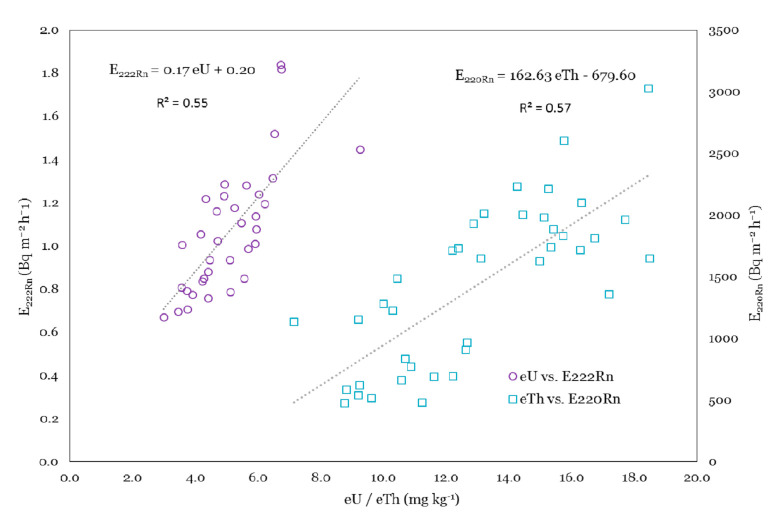
^222^Rn/^220^Rn exhalation rates plotted against Tellus airborne eU and eTh.

In the previous sections, good correlations between mean eU and radon exhalation rates (based on classifications by geology) and also eTh with thoron exhalation rates (based on soil type grouping) were identified. Moreover, as mentioned before, the airborne radiometric data is available in a high resolution for most of Ireland. Considering these, in this section, the precision and feasibility of radiometric data in the estimation of radon/thoron exhalation rates at the sampling points have been evaluated. To achieve this, at the first step, a linear fit between the estimated radioelement activity concentrations (i.e., eU and eTh) and radon/thoron exhalation rates was obtained. As can be seen in Figure 5 significant correlations between the estimated eU and radon exhalation rate (R^2^ = 0.55) and also the estimated eTh and thoron exhalation rates (R^2^ = 0.57) were found.

**Figure 6:**
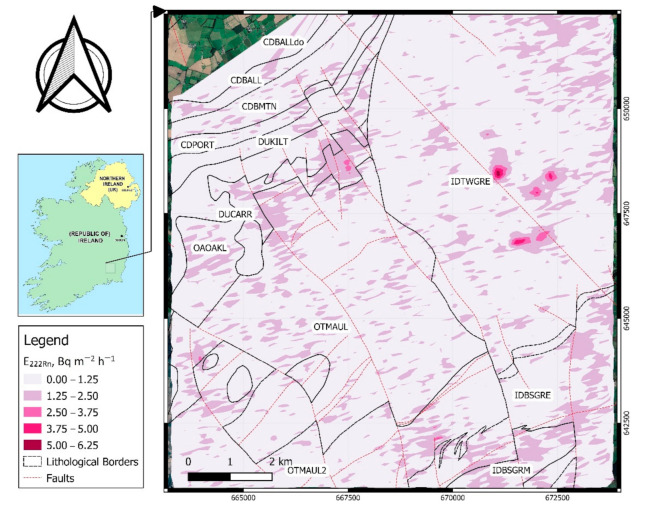
Modeled distribution of ^222^Rn exhalation rates overlain by geological boundaries.

**Figure 7:**
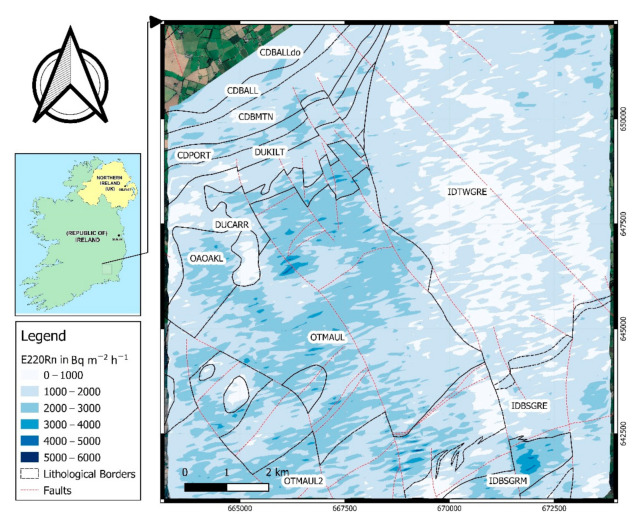
Distribution map of ^220^Rn exhalation rates overlain by soil type boundaries.

In the second step, based on the obtained relationships (Equations (4) and (5)) the radon and thoron exhalation rates were calculated for the area of airborne surveys. Figure 6 and Figure 7 show the spatial distributions of radon and thoron exhalation rates, respectively.

(4) $$E_{222\mathrm{Rn}}=0.17\cdot eU+0.20$$

(5) $$E_{222\mathrm{Rn}}=162.63\cdot eTh-679.60$$

As can be understood from Figure 6 variations in radon exhalation rates even within an individual geological unit can be seen, however, the highest values of exhalation rates occurred in granites with microcline phenocrysts (IDBSGRM) and green red-purple buff slate siltstone of Oakland’s formation (OAOAKL). In the case of thoron exhalation rate the estimated values are higher for IDBSGRM, OTMAUL (dark blue-grey slate, phyllite and schist), OTMAUL2 (Dark grey semi-pelitic, psammitic schist) and OAOAKL formations.

An important point in these two maps is that in a relatively small area heterogeneous geology occurred and this caused different radon exhalation rates. The variations are more obvious near geological boundaries. Figure 7 shows the distribution map thoron exhalation rates over the map of soil variations, as it can be understood from this map and also Table 4, the highest thoron exhalation rates are observed in soil associations of 0410a (peat over lithoskeletal acid igneous rock) and also fine and coarse loamy drift with siliceous stones (1100a and 1100c). The thoron exhalation rates in river alluvium (05RIV) are much lower when compared to other soil types. 

**Figure 8:**
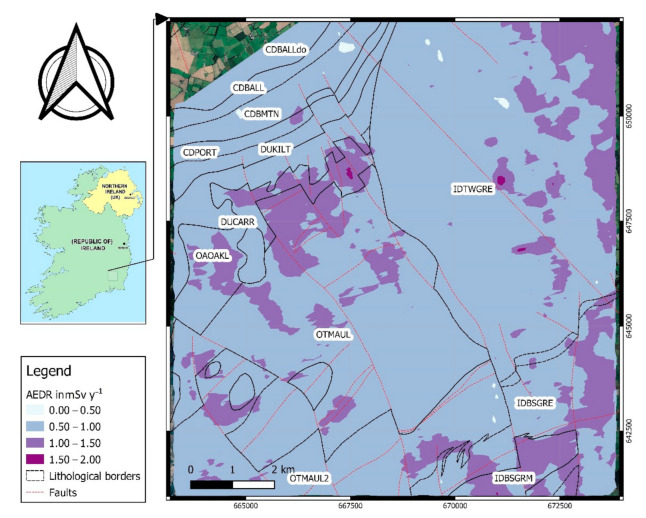
Map of estimated annual effective dose rates overlain by geological boundaries.

The annual effective dose rate (AEDR) in mSv y^−1^ was calculated using Equation (3) and the map of the distribution of annual effective dose rates was produced for the survey area, as shown in Figure 8. According to this map, the ADER for some areas exceeded the recommended level of 1 mSv y^−1^ for the general public by [44].

## 4. Discussion

The epidemiological effects of radon exposure in Ireland have been well studied and a correlation to annual numbers of lung cancer cases was estimated (approximately 270–280 ≈ 12% of the annual lung cancer incidence) [45]. According to the map of spatial variation in gamma radiation from the ground in Ireland produced by EPA [46], the highest values in the study area are associated with underlying granites. Furthermore, according to the same report, the highest mean indoor radon (123 Bq m^−3^) calculated for Irish counties was observed in County Carlow, where our study was conducted. The results reported by EPA are in agreement with the findings of our research. 

Although indoor radon has been thoroughly studied in some parts of the area under examination, few studies have addressed the dose coefficients for thoron and its decay products. The thoron exhalation map produced in this study shows that for some zones significant thoron release potential might be anticipated. Therefore, spatial and temporal thoron concentrations should be studied more. Generally, it is believed that thoron has a short half-life and may decay almost completely in indoor air, however, significant concentrations of its decay products (^212^Pb: half-life 10.6 h, ^212^Bi: half-life 1.01 h) remain. According to recent studies, it is estimated that exposure to air-containing thoron decay products at a concentration of 1 Bq m^−3^ gives rise to an average annual dose of approximately 750 µSv [46]. Therefore, it would be beneficial to include the dose from thoron (and its decay products) and highlight the possible adverse health effects of thoron and thoron progeny exposure in the definition of priority areas. Such an approach might be more important for houses without concrete foundations, or where the floors and/or walls are made of compacted earth, as is sometimes the case in rural areas in Ireland where older buildings have been renovated for residential use.

Another important point is that, according to the radon and thoron potential maps, although most of the areas with high radon potential also contain elevated thoron potential, there are radon priority areas that did not show high thoron release potentials. As an example, there is a small area in the central right side of Figure 6, where the radon exhalation potential is abnormally high but the thoron release potential for that area is low. This suggests a decoupling of radon and thoron in some areas, but not in others. It is suggested to carry out further in situ soil gas radon testing as well as indoor radon monitoring to achieve a better and more accurate view of radon potentials in that area.

## 5. Conclusions

Natural radioactivity levels and radon/thoron exhalation rates of twelve geological formations and ten soil types were analyzed using available data on indoor concentration, airborne radiometric and stream sediment geochemistry together with laboratory testing of radon and thoron activities and in situ dose rate measurements. In general, the occurrence of higher radon-related values (i.e., indoor radon, eU, U and radon exhalation rate) was associated with granitic bedrock and Oakland’s formation sedimentary rocks. The geometric mean (GM) indoor radon values of most of the studied geological units were found to be higher than the GM national concentration (57 Bq m^−3^). The mean radionuclide activity values (i.e., eU, eTh and K_air_) obtained were also found to be higher than worldwide averaged levels. For some parts of the study area where the ambient dose rate exceeds the average gamma dose rates measured for Ireland (i.e., 0.055 µGy h^−1^ indoors and 0.040 µGy h^−1^ outdoors) additional exposure risk should be considered. In general, the results of this study represent that a relatively high risk coming from radon/thoron and naturally occurring radioelements might be expected for some parts within the study area. The importance of thoron progeny measurements for a correct estimate of the effective dose was also highlighted. Geology was found to be the controlling factor to distinguish between the radon-related behaviors of different rock types. Using a soil type classification system, better correlations were found between thoron exhalation rates and the concentration of parent element Th. Among the geochemical elements analyzed for stream sediment samples (i.e., K_2_O, U, Th, Zr, Y, CaO, SiO_2_, Al_2_O_3_, MgO, and Fe_2_O_3_) the strongest correlations were found between U and Th with MgO and also between K_2_O with CaO, Al_2_O_3,_ and Y. Airborne estimated values (i.e., eU and eTh) were found as a promising tool for prediction of radon/thoron potentials. This method can be considered as a supplementary or even cross-validation for radon maps prepared based solely on indoor radon values. The maps produced in this research can be used to assess radon risk in both uninhabited and urban areas, as it is not dependent on the availability of indoor radon measurements. The method presented in this research can be applied to the other parts of Ireland or other countries where airborne data and geological and soil map data are available.

## Figures and Tables

**Table 1:** Summary of indoor radon, airborne radiometric, gamma dose and radon exhalation rates statistics for bedrock geologies. Please refer to Figure 1 for the description of bedrock codes.

Geology	Indoor Radon		Airborne Radiometric Data					Ambient Dose and Exhalation Rates (i.e., ADR and E)			
Bedrock Code	N. Measurements	Geometric Mean	N. Measurements	K_air_	eTh	eU	ADR_air_ (nGy h^−1^)	N. Measurements	ADR	E^222^Rn	E^220^Rn
		(Bq m^−3^)		(%)	(mg kg^−1^)				(μSv h^−1^)	(Bq m^−2^ h^−1^)	
CDBALL	1348	50	127	2.33	13.47	5.16	108.73	3	0.14	0.91	1521
CDBALLdo	132	72	48	2.26	13.74	4.63	106.38	3	0.17	1.12	1460
CDBMTN	118	57	293	2.55	13.53	5.04	112.29	3	0.16	1.18	1908
CDPORT	27	70	149	3.38	15.18	4.88	130.35	3	0.22	0.91	1797
DUCARR	101	69	471	4.04	12.10	4.42	133.39	4	0.19	0.91	2038
DUKILT	158	76	214	3.52	13.74	4.56	128.32	3	0.17	1.02	2016
IDBSGRE	46	188	765	4.87	12.92	5.31	153.97	6	0.20	0.94	746
IDBSGRM	2	366	177	5.15	18.25	6.17	178.43	3	0.21	1.52	1430
IDTWGRE	312	117	3220	4.83	11.43	5.10	147.37	4	0.18	1.10	1313
OAOAKL	226	119	468	4.60	16.09	5.79	160.33	3	0.22	1.29	2187
OTMAUL	387	71	2302	4.24	16.40	4.85	148.54	4	0.18	0.61	1302
OTMAUL2	29	120	1140	4.07	15.74	4.82	143.59	6	0.18	0.82	789

**Table 2:** Summary of soil geochemistry for grouped bedrock geologies. Numbers specified with * represent only the value of a single measurement. Please refer to Figure 1 for the description of bedrock codes.

Geology	Stream Sediment Geochemical Data										
Bedrock	N. Measurements	MgO	Al_2_O_3_	SiO_2_	K_2_O	CaO	Fe_2_O_3_	Y	Zr	Th	U
		(%)								(mg kg^−1^)	
CDBALL	53	1.55	9.26	46.55	1.44	4.90	4.81	21.21	303.16	6.73	3.54
CDBALLdo	2	1.60	10.30	59.20	1.90	2.65	4.33	20.45	815.00	8.45	2.60
CDBMTN	5	1.22	10.52	59.10	2.29	1.61	4.01	22.84	489.86	8.40	2.74
CDPORT	1 *	1.40	16.30	60.60	4.14	0.42	5.03	36.40	339.40	10.20	4.20
DUCARR	11	1.22	12.95	60.05	3.66	0.82	4.83	32.06	488.30	9.65	3.85
DUKILT	6	1.25	13.45	59.90	3.16	0.90	5.27	31.43	484.85	9.50	3.15
IDBSGRE	35	0.70	14.41	55.64	3.71	0.77	2.69	21.45	212.45	17.37	40.10
IDBSGRM	0	-	-	-	-	-	-	-	-	-	-
IDTWGRE	117	1.15	10.86	54.52	2.49	4.23	2.77	22.99	352.31	16.01	11.96
OAOAKL	104	1.51	17.91	57.84	2.89	0.45	6.73	30.66	266.38	10.57	2.89
OTMAUL	152	1.54	16.36	53.27	2.87	0.78	6.64	32.98	284.32	10.79	3.07
OTMAUL2	23	1.37	15.18	56.07	3.25	0.57	5.73	32.00	327.55	9.52	4.28

**Table 3:** Pearson’s correlation matrix of indoor radon, airborne radiometric data, geochemical data, gamma dose and radon exhalation rates categorized based on geology type.

	GM_Rn_	E_222Rn_	eU	U	E_220Rn_	eTh	Th	K_air_	K_2_O	MgO	Al_2_O_3_	SiO_2_	CaO	Fe_2_O_3_	Y	Zr	ADR-Air	ADR
GM_Rn_	1.00																	
E_222Rn_	**0.62**	1.00																
eU	**0.77**	**0.69**	1.00															
U	**0.84**	−0.05	0.32	1.00														
E_220Rn_	−0.32	0.31	−0.08	−0.59	1.00													
eTh	0.53	0.25	0.56	−0.35	−0.05	1.00												
Th	**0.83**	0.01	0.32	**0.82**	−0.48	−0.33	1.00											
K_air_	**0.67**	0.19	0.50	0.50	−0.28	0.27	**0.79**	1.00										
K_2_O	0.40	−0.27	−0.14	0.32	−0.03	0.15	0.40	0.58	1.00									
MgO	**−0.69**	−0.05	−0.09	**−0.85**	0.34	0.54	**−0.76**	−0.51	−0.47	1.00								
Al_2_O_3_	0.36	−0.22	0.28	0.06	0.03	**0.72**	0.21	**0.61**	**0.70**	0.02	1.00							
SiO_2_	0.00	0.34	−0.33	−0.12	0.42	0.01	0.01	0.05	0.56	−0.16	0.30	1.00						
CaO	−0.25	0.16	0.07	−0.06	−0.11	−0.54	−0.07	−0.37	**−0.79**	0.17	**−0.81**	**−0.65**	1.00					
Fe_2_O_3_	−0.35	−0.24	0.03	**−0.64**	0.35	**0.84**	−0.56	−0.02	0.07	**0.70**	**0.60**	0.02	−0.44	1.00				
Y	−0.16	−0.39	−0.24	−0.39	0.30	0.56	−0.17	0.31	**0.69**	0.24	**0.76**	0.40	**−0.70**	**0.68**	1.00			
Zr	−0.47	0.31	**−0.60**	−0.40	0.27	−0.26	−0.43	**−0.62**	−0.34	0.31	−0.51	0.45	0.16	−0.13	−0.26	1.00		
ADR-air	**0.78**	0.32	**0.67**	0.42	−0.24	0.53	**0.69**	**0.95**	0.56	−0.36	**0.75**	0.03	−0.46	0.18	0.39	**−0.69**	1.00	
ADR	0.46	0.26	0.39	0.20	0.12	0.44	0.41	**0.67**	**0.80**	−0.19	**0.84**	0.57	**−0.73**	0.23	**0.61**	−0.32	**0.71**	1.00

(GM_Rn_: Geometric mean of indoor Rn calculated with respect to the bedrock classes, E: exhalation rate, eU/eTh: equivalent uranium and thorium contents from airborne radiometric, ADR_air_: air absorbed dose rate, ADR: ambient dose rate and the reminder of symbols collate the geochemical parameters analyzed by XRF). The bold numbers indicate a statistically significant correlation.

**Table 4:** Summary of indoor radon, airborne radiometric, gamma dose and radon exhalation rates statistics for soil types. Numbers with specified * represent only the value of a single measurement. Please refer to Figure 2 for the description of soil associations.

Soil Type	Indoor Radon		Airborne Radiometric Data					Ambient Dose and Exhalation Rates			
Association	N. Measurements	Geometric Mean	N. Measurements	K_air_	eTh	eU	ADR_air_ (nGy h^−1^)	N. Measurements	ADR	E_222Rn_	E_220Rn_
		(Bq m^−3^)		(%)	(mg kg^−1^)				(μSv h^−1^)	(Bq m^−2^ h^−1^)	
0410a	1 *	729	719	4.43	16.22	4.73	152.05	0	-	-	-
05RVI	0	-	400	3.67	10.68	4.89	125.74	5	0.19	0.75	896
0660e	5	296	141	5.48	12.99	5.80	165.41	0	-	-	-
0960c	4	131	120	3.81	11.76	3.90	126.16	0	-	-	-
1000c	13	105	129	4.63	11.75	4.46	140.02	0	-	-	-
1000x	95	99	691	2.77	14.03	5.02	117.23	15	0.17	1.03	1740
1100a	36	194	2673	4.13	15.89	4.85	145.22	3	0.18	0.71	1648
1100c	4	105	892	4.42	14.94	5.20	151.12	6	0.21	1.07	2138
1100h	76	304	3636	4.92	12.28	5.21	151.93	13	0.20	1.15	1109
Urban	16	319	89	3.90	11.19	4.37	126.43	3	0.17	0.79	706

**Table 5:** Summary of soil geochemistry for grouped soil associations. Numbers with specified * represent only the value of a single measurement. Please refer to Figure 2 for the description of soil associations.

Soil Type	Stream Sediment Geochemical Data										
Association	N. Measurements	MgO	Al_2_O_3_	SiO_2_	K_2_O	CaO	Fe_2_O_3_	Y	Zr	Th	U
		(%)								(mg kg^−1^)	
0410a	1 *	1.70	18.60	59.30	3.37	0.32	5.95	38.20	255.00	8.80	2.80
05RVI	29	1.40	14.44	54.68	3.03	2.08	4.77	29.24	276.73	11.55	8.26
0660e	2	0.70	13.90	55.30	3.73	1.25	2.23	26.10	292.15	33.40	39.05
0960c	0	-	-	-	-	-	-	-	-	-	-
1000c	3	2.20	8.67	55.17	2.22	5.17	1.63	18.37	386.90	8.53	5.77
1000x	1 *	1.40	16.30	60.60	4.14	0.42	5.03	36.40	339.40	10.20	4.20
1100a	10	1.81	15.61	54.66	3.01	1.44	5.51	34.08	265.63	8.31	3.32
1100c	1 *	1.90	21.20	56.00	3.77	0.18	6.72	39.80	204.80	8.60	3.40
1100h	8	1.35	13.55	52.94	2.99	2.02	3.91	26.49	266.73	12.69	14.91
Urban	0	-	-	-	-	-	-	-	-	-	-

**Table 6:** Pearson’s correlation matrix of indoor radon, airborne radiometric, geochemical data, gamma dose and radon exhalation rates categorized based on soil type. (GM_Rn_: Geometric mean of indoor Rn calculated with respect to the soil classes, E: exhalation rate, eU/eTh: equivalent uranium and thorium contents from airborne radiometric, ADR_air_: air absorbed dose rate, ADR: ambient dose rate and the reminder of symbols collate the geochemical parameters analyzed by XRF). The bold numbers indicate a statistically significant correlation.

	GM_Rn_	E_222Rn_	eU	U	E_220Rn_	eTh	Th	K_air_	K_2_O	MgO	Al_2_O_3_	SiO_2_	CaO	Fe_2_O_3_	Y	Zr	Adr Air	ADR
GM_Rn_	1.00																	
E_222Rn_	−0.20	1.00																
eU	0.08	**0.73**	1.00															
U	0.05	0.40	**0.82**	1.00														
E_220Rn_	**−0.93**	0.39	**0.62**	**−0.74**	1.00													
eTh	0.08	**0.73**	0.08	**0.82**	**0.62**	1.00												
Th	0.07	0.34	**0.83**	**0.98**	**−0.82**	**0.83**	1.00											
K_air_	0.29	0.26	0.45	**0.64**	−0.06	0.45	0.56	1.00										
K_2_O	0.02	0.44	0.61	0.18	**0.69**	**0.61**	0.30	−0.30	1.00									
MgO	−0.18	−0.20	**−0.84**	**−0.83**	**0.75**	**−0.84**	**−0.85**	−0.22	−0.52	1.00								
Al_2_O_3_	0.25	0.24	0.23	−0.28	**0.88**	0.23	−0.19	−0.23	**0.71**	−0.01	1.00							
SiO_2_	0.24	0.14	−0.17	−0.32	0.50	−0.17	−0.18	−0.58	0.59	0.04	0.42	1.00						
CaO	−0.31	−0.35	−0.47	0.02	**−0.93**	−0.47	−0.09	0.24	**−0.88**	0.37	**−0.91**	−0.48	1.00					
Fe_2_O_3_	0.20	−0.10	−0.10	**−0.60**	**0.84**	−0.10	−0.53	−0.46	0.47	0.23	0.90	0.35	**−0.76**	1.00				
Y	0.24	0.06	0.08	−0.43	**0.94**	0.08	−0.32	−0.44	**0.70**	0.05	**0.96**	0.57	**−0.90**	**0.94**	1.00			
Zr	−0.32	−0.08	−0.36	0.05	−0.30	−0.36	0.04	−0.18	−0.38	0.11	**−0.81**	0.15	**0.70**	**−0.74**	**−0.67**	1.00		
ADR air	0.39	0.35	**0.63**	0.56	0.34	**0.63**	0.52	**0.91**	−0.05	−0.23	0.10	−0.39	−0.09	−0.17	−0.10	−0.42	1.00	
ADR	−0.13	0.52	**0.72**	0.37	0.30	**0.72**	0.18	**0.67**	−0.22	0.24	0.37	**−0.58**	0.06	0.21	−0.08	**−0.84**	**0.71**	1.00

## Data Availability

Publicly available datasets were analyzed in this study. This data can be found here: www.gsi.ie (accessed on 26 November 2019) and www.epa.ie (accessed on 26 November 2019).
